# Pro-oxidant/antioxidant balance controls pancreatic *β*-cell differentiation through the ERK1/2 pathway

**DOI:** 10.1038/cddis.2014.441

**Published:** 2014-10-23

**Authors:** E Hoarau, V Chandra, P Rustin, R Scharfmann, B Duvillie

**Affiliations:** 1 INSERM, U1016, Institut Cochin, Paris, France; 2 Université Paris Descartes, Sorbonne Paris Cité, Faculté de Médecine, Paris, France; 3 INSERM U676, Hopital Robert Debre, Paris, France

## Abstract

During embryogenesis, the intrauterine milieu affects cell proliferation, differentiation, and function by modifying gene expression in susceptible cells, such as the pancreatic *β*-cells. In this limited energy environment, mitochondrial dysfunction can lead to overproduction of reactive oxygen species (ROS) and to a decline in *β*-cell function. In opposition to this toxicity, ROS are also required for insulin secretion. Here we investigated the role of ROS in *β*-cell development. Surprisingly, decreasing ROS production *in vivo* reduced *β*-cell differentiation. Moreover, in cultures of pancreatic explants, progenitors were highly sensitive to ROS stimulation and responded by generating *β*-cells. ROS enhanced *β*-cell differentiation through modulation of ERK1/2 signaling. Gene transfer and pharmacological manipulations, which diminish cellular ROS levels, also interfered with normal *β*-cell differentiation. This study highlights the role of the redox balance on *β*-cell development and provides information that will be useful for improving *β*-cell production from embryonic stem cells, a step in cell therapy for diabetes.

Reactive oxygen species (ROS) exist in all aerobic cells, and their level is controlled by a balance between pro-oxidants and antioxidants. ROS include the superoxide radical, hydrogen peroxide (H_2_O_2_), singlet oxygen, and the hydroxyl radical. An excess of ROS leads to oxidative stress, one of the most important regulatory mechanisms for cancer cells, and cancer stem cells.^1^ During the past decade, the cytotoxic effects of ROS have been implicated in various human diseases including cancer and Parkinson's disease.^2^ However, low physiological levels of ROS have also been reported recently to operate as signaling molecules.^3^ Indeed, depending on the context, ROS can modulate cell proliferation,^4^ survival,^5^ and differentiation.^6^

The endocrine pancreas is exceptionally sensitive to variations in intracellular levels of ROS. Indeed, ROS-inactivating enzymes are expressed at very low levels in pancreatic *β*-cells, rendering them highly sensitive to oxidative stress.^7,8^ Hyperglycemia is a cause of oxidative stress-related damage in a number of cell types, including neurons, retinal cells, vascular endothelium,^9^ and pancreatic *β*-cells. Persistent hyperglycemia in diabetes increases ROS production by glucose autoxidation,^10^ activation of protein kinase C (PKC), and increased flux through the hexosamine pathway.^9^ In human type II diabetes, the link between oxidative stress and *β*-cell dysfunction is well established.^11,12^ However, despite these toxic effects, recent findings have shed light on the necessity of ROS for *β*-cell function.^13^

Redox status has been suggested to modulate stemness and lineage commitment in several precursor cell types.^14^ In this study, we researched the effects of ROS on pancreatic progenitor cells. Pancreas development is governed by a hierarchy of transcription factors.^15^ In the mouse, embryonic pancreatic progenitors arise at ∼E9 from the primitive intestine, expressing the homeodomain transcription factor PDX1 (pancreatic and duodenal homeobox 1).^16^ PDX1 is necessary for the morphogenesis and differentiation of the pancreatic buds in both rodents and humans.^17,18^ Upon FGF10 signaling, the pancreatic epithelium grows.^19^ Endocrine progenitors that transiently express the transcription factor neurogenin 3 (NGN3) will give rise to all of the pancreatic endocrine cell types.^20^

Here, we investigated the role of ROS in endocrine pancreas development. We first showed that treatment of pregnant rats with *N*-acetyl-cysteine (NAC) decreases *β*-cell differentiation in their progeny. We then investigated by which mechanism ROS control *β*-cell differentiation. Using culture models, we showed that H_2_O_2_ enhances the generation of *β*-cells in the rat embryonic pancreas. Using both chemical and genetic approaches, we next demonstrated that overexpression of catalase decreases *β*-cell differentiation. This effect of catalase was mimicked by the antioxidant NAC or the decoupling agent carbonyl cyanide *m*-chlorophenyl hydrazone (CCCP) that both decrease ROS production. We finally found that the ERK1/2 pathway was activated by H_2_O_2_ and, interestingly, the effect of H_2_O_2_ on *β*-cell differentiation was blunted by an inhibitor of the ERK1/2 pathway. Altogether, these data demonstrate that pancreatic endocrine cell development is tightly dependent of ROS.

## Results

### ROS are required for *β*-cell development *in vivo*

Several stem cell types are sensitive to ROS signals.^21,22^ Here, we investigated whether endocrine cell development is modulated by ROS in the embryonic pancreas *in vivo*. First, we quantified the expression of antioxidant enzyme genes in the embryonic and adult pancreases and livers (Supplementary Figure S1). Catalase and glutathione peroxidase (Gpx) are responsible for the degradation of H_2_O_2_. At the adult stage, their mRNA levels were reduced in pancreases compared with livers (Supplementary Figure S1) and were even lower in embryonic (E13.5) pancreases. These data suggest that developing embryonic pancreas is highly susceptible to oxidative stress because of low expression of these ROS-scavenging enzymes. Moreover, if not eliminated by Catalase or Gpx, H_2_O_2_ can activate signaling pathways,^23^ potentially involved in cell division and differentiation within a narrow window of development.^24^

**Figure 1:**
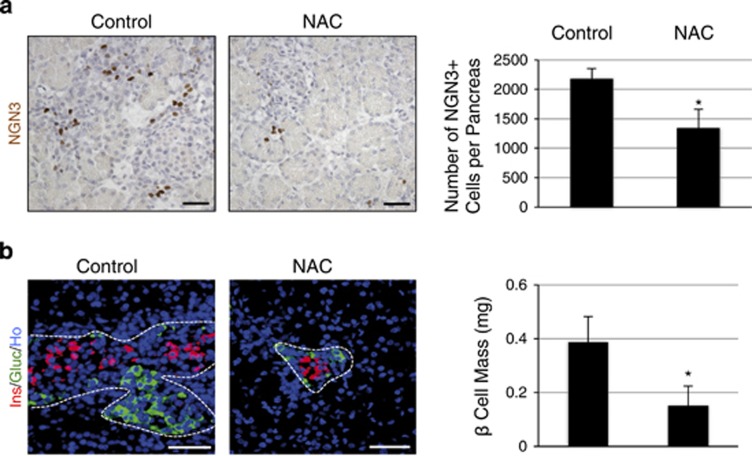
Antioxidant NAC decreases *β*-cell differentiation *in vivo.* Pregnant rats were treated with 10 mM NAC from 13.5 days post coitum. Embryonic pancreases were analyzed at E20.5 (**a**) NGN3 expression (in brown) was detected by immunohistochemistry, and the number of NGN3-positive cells was quantified. (**b**) Islets were detected in treated and control embryonic pancreases using anti-insulin (red) and anti-glucagon antibodies (green). Nuclei were stained with Hoechst 33342 (blue). The *β*-cell mass was then calculated. Each point represents the mean±S.E.M. of three individual data pools. *P<0.05. Scale bar: 50 *μ*m

Therefore, we tested whether modulating the level of ROS would affect pancreatic endocrine development. We treated pregnant rats at 13.5 days post coitum with NAC, a precursor of the biologic antioxidant gluthatione, and examined pancreatic development in their progeny at E20.5. The morphology of the treated pancreas was normal (Figure 1 and Supplementary Figure S2). The presence of acinar cells, and also *α*-, *β*-, and *δ*-cells, was detected using anti-amylase, anti-glucagon, anti-insulin, and anti-somatostatin antibodies, respectively (Supplementary Figure S2 and Figure 1b). The endocrine progenitors were next detected using anti-NGN3 antibodies (Figure 1a). We observed a decreased number of NGN3-positive cells (1.6-fold) in the pancreases from fetuses treated with NAC compared with controls (Figure 1a), suggesting a reduction of endocrine differentiation. To confirm this hypothesis, we quantified the *α*-cell and *β*-cell masses. We found a 3.3-fold decrease of the *α*-cell mass and a 2.5-fold reduction of the *β*-cell mass in the pancreases treated with NAC (Supplementary Figure S2 and Figure 1b). These results thus implicate ROS in *β*-cell differentiation.

### ROS enhance *β*-cell differentiation in embryonic pancreases

**Figure 2:**
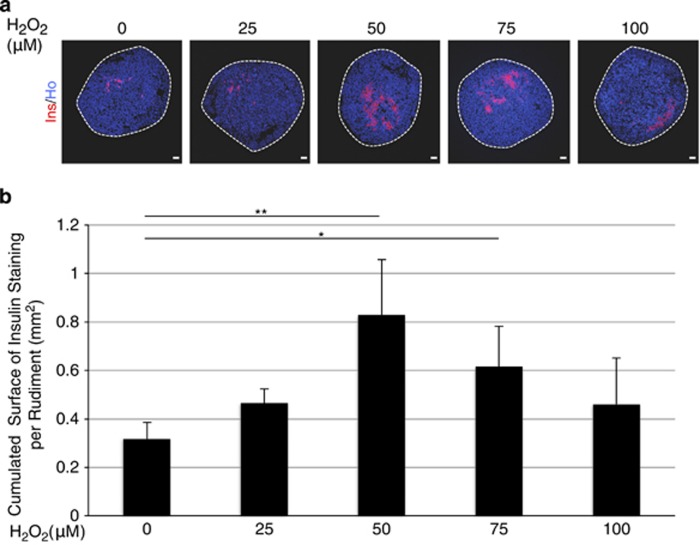
Effect of H_2_O_2_ on *β*-cell development. (**a**) E13.5 rat pancreases were cultured for 7 days with H_2_O_2_ (0–100 *μ*M). At day 7, anti-insulin antibody (red) was used to detect *β*-cell development. Nuclei were stained with Hoechst 33342 (blue). Scale bar: 50 *μ*m. (**b**) The absolute surface area occupied by insulin-positive cells was quantified. Each point represents the mean±S.E.M. of three individual data pools. *P<0.05; **P<0.01

To investigate how ROS regulate pancreatic *β*-cell development, we cultured E13.5 rat pancreases with various concentrations of ROS. Instead of manipulating superoxide levels, which is a difficult task because of its short half-life,^25^ we used H_2_O_2_. Indeed, H_2_O_2_ is diffusible and penetrates efficiently into the cells.^23^ Using polarography we first determined H_2_O_2_ stability in our culture conditions by measuring its decay over time^26^ (Supplementary Figure S3). We found that 50% of the initial quantity of H_2_O_2_ remains present after 4 h in the culture medium. Next, pancreatic explants were treated with increasing concentrations (0–100 *μ*M) of H_2_O_2._ We first attempted to apply H_2_O_2_ every day during the culture period, but such treatment became toxic after 3 days (data not shown). Therefore, in the following experiments, H_2_O_2_ was added only at the beginning of the culture. When H_2_O_2_ treatment was applied to cultured pancreases, expression of the oxidative stress response genes, *nuclear-factor E2 related Factor 2* (*Nrf2*) and *heme oxygenase 1* (*HO-1*) was upregulated (1.6-fold and 2-fold respectively, Supplementary Figure S4). Interestingly, after 7 days in culture, the development of *β*-cells was increased with 50 and 75 *μ*M H_2_O_2_, the surface occupied by insulin-positive cells being 2.6- and 2-fold larger than controls (Figures 2a and b). At two other concentrations, 25 and 100 *μ*M, we also observed a slight increase of *β*-cell development. To confirm this effect of H_2_O_2_, we also quantified insulin content in pancreases that develop with H_2_O_2_ treatment. We found a 1.6-fold increase in pancreases cultured 7 days with H_2_O_2_ compared with controls (Supplementary Figure S5). The transcription factor PDX1, which is expressed in mature differentiated *β*-cells,^27^ was present in all the insulin-positive cells (Supplementary Figure S6), confirming that H_2_O_2_ enhances *β*-cell development.

**Figure 3:**
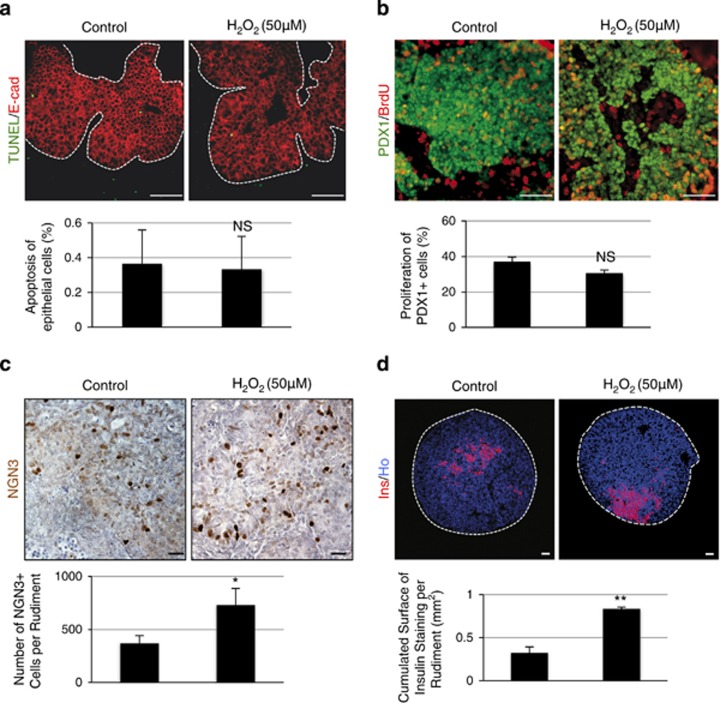
H_2_O_2_ induces *β*-cell differentiation. (**a**) Apoptotic cells (in green) were detected using a TUNEL reaction assay in pancreases cultured for 1 day with or without 50 *μ*M H_2_O_2_. The epithelial cells were detected using an anti-E-cadherin antibody (in red). Note the absence of apoptotic cells within the epithelium. Next, apoptosis percentage was calculated. Scale bar: 50 *μ*m. (**b**) BrdU was added 1 h before the end of culture. Proliferation of early PDX1^+^ progenitors was observed in pancreases cultured for 1 day with or without 50 *μ*M H_2_O_2_ (PDX1 in green and BrdU in red). Proliferation percentage was also quantified. Scale bar: 50 *μ*m. (**c**) On day 1, NGN3 expression (in brown) was detected by immunohistochemistry. For each pancreas, the number of NGN3^+^ cells was quantified. Scale bar: 25 *μ*m (**d**) On day 7, immunohistochemistry was performed to detect insulin (in red). Nuclei were stained with Hoechst 33342 (blue). The insulin-stained surface was measured under both conditions. Each point represents the mean±S.E.M. of three individual data pools. Scale bar: 50 *μ*m. *P<0.05; **P<0.01

We next sought to determine the mechanism by which H_2_O_2_ increases *β*-cell development. We tested whether H_2_O_2_ increases the survival or the proliferation of progenitor cells, or amplifies the differentiation of progenitor cells into *β*-cells. After 1 day of culture in the presence or in the absence of H_2_O_2_, the rate of apoptosis measured using a TUNEL reaction did not differ between the H_2_O_2_-treated pancreases and controls (Figure 3a). The *β*-cell proliferation measured following BrdU incorporation was similar in pancreases cultured with or without H_2_O_2_ (Figure 3b). Finally, we investigated the effect of H_2_O_2_ on *β*-cell differentiation. The number of NGN3-positive endocrine progenitor cells was increased by nearly twofold in pancreases cultured with H_2_O_2_ (Figure 3c), followed by an increased number of *β*-cells (Figure 3d). Thus, these results show that H_2_O_2_ controls *β-*cell differentiation. To confirm this result, we cultured pancreases with glucose oxidase (GOx), an enzyme that oxidizes glucose into H_2_O_2_ and D-glucono-lactone. In the presence of GOx, H_2_O_2_ is continuously generated in the medium. We found that the number of NGN3-positive cells was increased 2.2-fold in the presence of GOx compared with controls (Supplementary Figure S7). These data thus demonstrate that ROS have a positive and nontoxic effect on *β-*cell development.

### Increasing catalase levels alters Ngn3 expression

**Figure 4:**
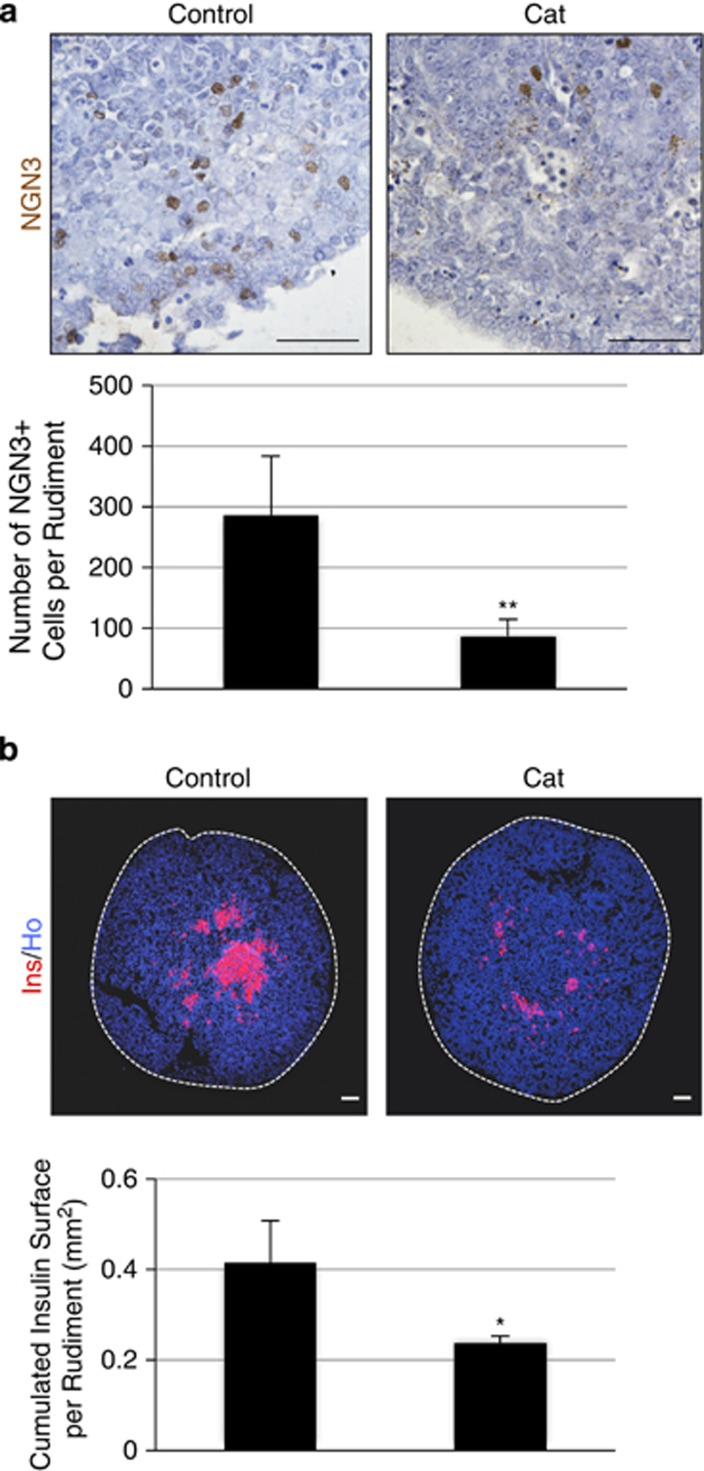
Exogenous catalase reduces *β*-cell differentiation. E13.5 pancreases were cultured for 1 to 7 days with or without catalase at 300 U/ml. (**a**) After 1 day of culture, NGN3 expression was detected by immunohistochemistry (in brown). The absolute number of NGN3^+^ cells was quantified. (**b**) After 7 days, *β*-cell area (in red) was detected by immunohistochemistry and quantified. Nuclei were stained with Hoechst 33342 (blue). Each point represents the mean±S.E.M. of three individual data pools. Scale bar: 50 *μ*m. *P<0.05; **P<0.01

In order to control H_2_O_2_ production, we manipulated catalase expression that catalyzes the degradation of H_2_O_2_ into water and oxygen. Catalase level was increased by using both pharmacological and genetic methods. E13.5 pancreases were first cultured for 1 day with exogenous catalase. We observed a 3.3-fold decrease in the number of NGN3-expressing cells compared with controls (Figure 4a). Consistent with this result, we observed a 1.75-fold reduction of the insulin-staining surface in pancreases treated with catalase (Figure 4b).

**Figure 5:**
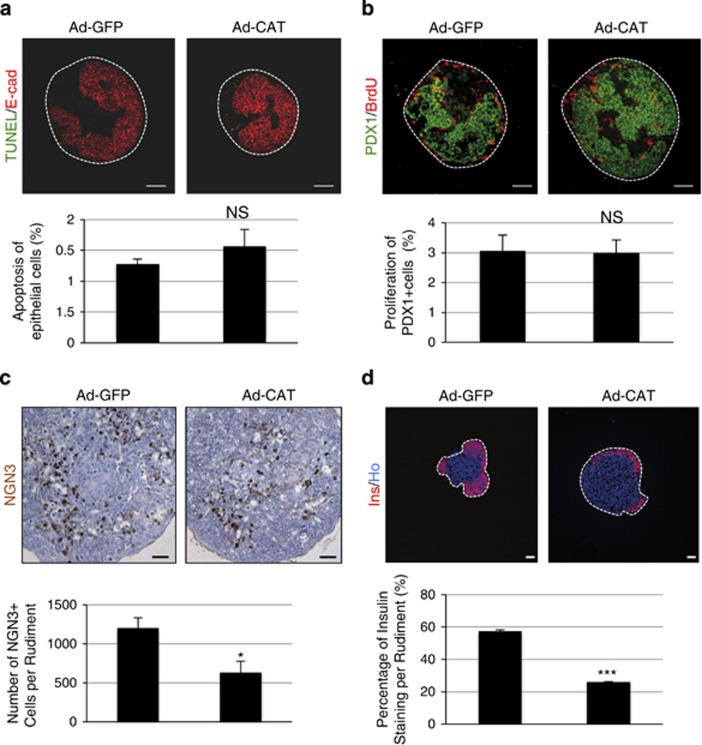
Catalase overexpression decreases NGN3 expression in the pancreatic epithelium. E13.5 rat pancreatic epithelia were infected by adenovirus coding either for GFP (controls) or for catalase and cultured for 1, 3, or 7 days. (**a**) Apoptotic cells (in green) were detected using a TUNEL reaction assay at culture day 1. The epithelial cells were detected using an anti-E-cadherin antibody (in red). Next, apoptosis percentage of epithelial cells was calculated. Scale bar: 50 *μ*m. (**b**) Proliferation of early PDX1^+^ progenitors was detected by immunohistochemistry (PDX1 in green and BrdU in red) at culture day 1. Proliferation percentage was also quantified. Scale bar: 50 *μ*m. (**c**) On day 3, NGN3 expression (in brown) was detected by immunohistochemistry and the number of NGN3^+^ cells per epithelium was quantified. Scale bar: 25 *μ*m. (**d**) After 7 days, *β*-cell area (in red) was detected by immunohistochemistry and quantified. Nuclei were stained with Hoechst 33342 (blue). Each point represents the mean±S.E.M. of three individual data pools. Scale bar: 50 *μ*m. *P<0.05; ***P<0.001

We next increased endogenous catalase expression using adenoviral transduction. E13.5 pancreatic epithelia were infected with adenoviruses coding either for catalase (Ad-Cat) or green fluorescent protein (Ad-GFP, control). Pancreases infected with Ad-GFP were negative for catalase protein expression. Following Ad-Cat transduction, catalase expression was found in many pancreatic epithelial cells (Supplementary Figure S8). However, some cells at the center of the rudiments were not reached by the virus. Therefore, a milder effect of Ad-Cat on *β*-cell differentiation was expected as compared with the exogenous catalase treatment. Infection with Ad-Cat did not modify epithelial progenitor cell apoptosis as measured by TUNEL assay, nor progenitor cell proliferation as measured by quantification of BrdU incorporation (Figures 5a and b). On the other hand, the number of NGN3-positive endocrine progenitor cells was decreased by 1.6-fold in pancreases infected by Ad-Cat compared with pancreases infected by Ad-GFP (Figure 5c). Finally, *β*-cell development was decreased in pancreases infected with Ad-Cat *versus* controls (Figure 5d). Altogether, these data demonstrate that catalase levels regulate *β*-cell development.

### Effects of ROS modulators on endocrine cell development

**Figure 6:**
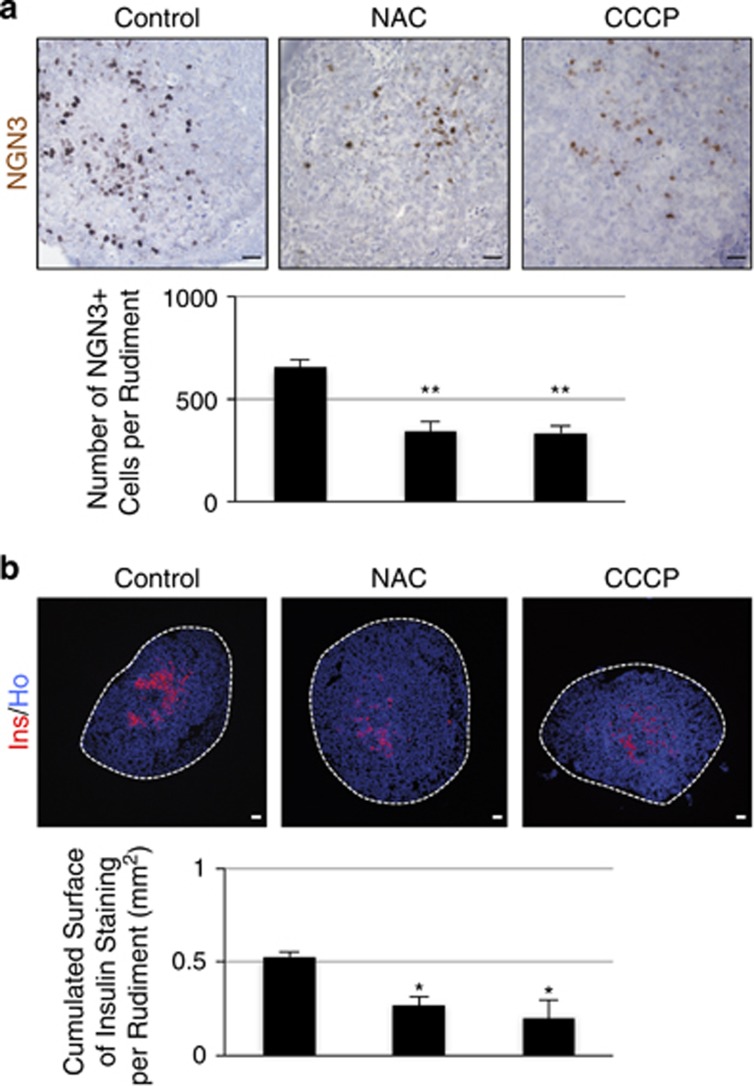
*N*-acetyl-cysteine and the mitochondrial agent CCCP alter *β*-cell differentiation. E13.5 rat pancreases were cultured for 1 and 7 days with or without NAC at 10 *μ*M or CCCP at 1 *μ*M. (**a**) After 1 day, NGN3 expression (in brown) was detected by immunohistochemistry. The number of NGN3^+^ cells per epithelium was quantified. Scale bar: 25 μm. (**b**) On day 7, *β*-cell area (in red) was detected by immunohistochemistry and quantified. Nuclei were stained with Hoechst 33342 (blue). Each point represents the mean±S.E.M. of three individual data pools. Scale bar: 50 *μ*m. *P<0.05; **P<0.01

To further determine the effects of ROS modulators on endocrine cell differentiation, we cultured embryonic pancreases in the presence of the antioxidant NAC. This treatment decreased nearly twofold the number of NGN3-positive endocrine progenitor cells, followed by a decrease of *β*-cell development (Figures 6a and b).

Mitochondria are the major site of ROS production.^28^ To determine the implication of mitochondria in *β*-cell development, we used the mitochondrial decoupling agent CCCP. This compound is known to decrease ROS levels. We found that CCCP reduced the number of NGN3-positive cells at day 1 and the insulin-staining surface at day 7 (Figures 6a and b). We thus conclude that endogenous ROS are mandatory for *β*-cell differentiation. Moreover, the effects obtained with CCCP suggest that in this process, ROS have probably a mitochondrial origin.

### Regulation of *β*-cell development by ROS levels depends on ERK1/2 signaling

**Figure 7:**
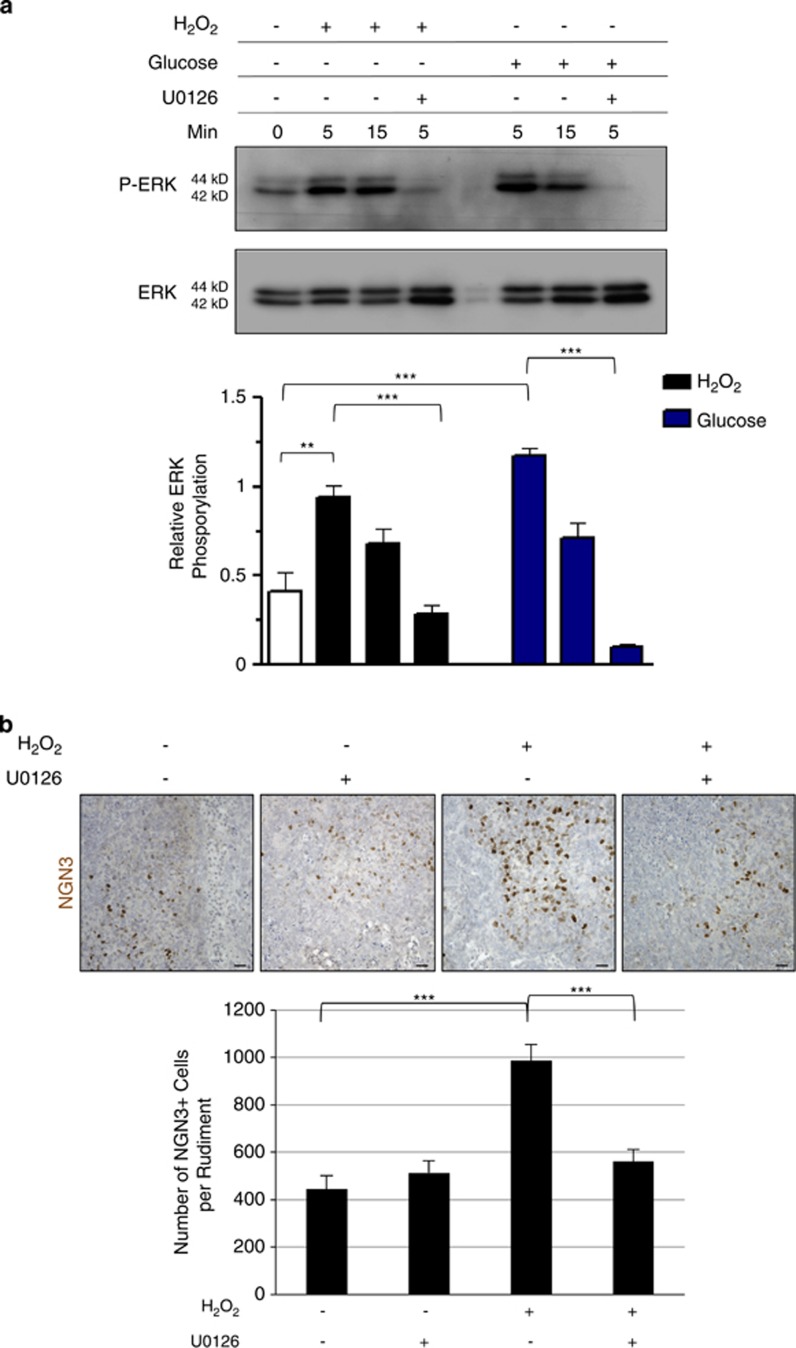
Activation of ERK1/2 phosphorylation by H_2_O_2_ is required for the proper development of *β*-cells. (**a**) E13.5 rat pancreases were cultured for 0, 5, or 15 min with or without 50 *μ*M H_2_O_2_ in association or not with U0126. Glucose 20 mM was used as a positive regulator of ERK1/2. Protein extracts from cultured pancreases were analyzed by western blot to quantify P-ERK1/2. Total ERK is used as loading control. Relative ERK Phosphorylation was also quantified for each condition. (**b**) E13.5 rat pancreases were cultured with or without H_2_O_2_ at 50 *μ*M, in association or not with the ERK1/2 inhibitor U0126. For each condition, NGN3 expression (in brown) was detected by immunohistochemistry and the number of NGN3^+^ cells was quantified. Each point represents the mean±S.E.M. of three individual data pools. Scale bar: 25 μm. *P<0.05; **P<0.01, ***P<0.001

We next characterized the molecular mechanisms by which H_2_O_2_ increased *β*-cell development. We examined the mitogen-activated protein kinase (MAPK) ERK1/2 pathway that was recently shown to be sensitive to ROS production in several cell types.^29^ Pancreases were cultured with or without H_2_O_2_ (50 *μ*M) for 5 or 15 min. In parallel experiments, pancreases were treated with 20 mM glucose, a well-known inducer of ERK1/2 phosphorylation in *β*-cells and fetal islets.^30,31^ Western blot analysis showed that H_2_O_2_, similar to high glucose, induced the ERK1/2 pathway (Figure 7a). Such an induction of the ERK1/2 pathway by H_2_O_2_ was also observed by immunohistochemistry (Supplementary Figure S9).

To characterize the link between the ROS-activated ERK1/2 pathway and endocrine development, we used U0126, an inhibitor of ERK1/2 phosphorylation. When explants cultured with high glucose levels were treated with U0126, activation of ERK1/2 was abolished (Figure 7a and Supplementary Figure S9), validating the efficiency of the ERK1/2 inhibitor. We next compared the phosphorylation of ERK1/2 by H_2_O_2_ in the presence or absence of U0126. As expected, activation of the ERK1/2 pathway by H_2_O_2_ was blunted in the presence of U0126 (Figure 7a and Supplementary Figure S9). We next researched whether ERK1/2 activation by H_2_O_2_ is necessary for the induction of *β*-cell differentiation. For this, pancreases were treated with H_2_O_2_ in the presence or absence of U0126 and NGN3 expression was analyzed. Treatment of pancreases with H_2_O_2_ considerably increased the number of NGN3-expressing cells (Figure 7b). Interestingly, the repression of ERK1/2 by U0126 abolished the inductive effect of H_2_O_2_ on NGN3 expression (Figure 7b). Altogether, these results demonstrate that the effects of H_2_O_2_ on *β*-cell differentiation are mediated by the ERK1/2 pathway.

## Discussion

### ROS regulate pancreatic progenitor cell differentiation

Here we have demonstrated that ROS have a significant impact on pancreatic progenitor cell differentiation. This result was unexpected as ROS have been implicated in *β*-cell toxicity in other models. For example, uteroplacental insufficiency in rats leads to intrauterine growth retardation (IUGR) and disrupts the function of the electron transport chain in the fetal *β*-cell. This process induces increased production of ROS that in turn damages mitochondrial DNA and causes further production of ROS.^32^ These events are involved in a progressive loss of *β*-cell function and type II diabetes in the adult. This model of IUGR is thought to mimic type II diabetes in humans, with progressive defects in insulin secretion and insulin action before hyperglycemia. A difference between the IUGR model and the present study is that the increase of ROS occurred in differentiated *β*-cells in the IUGR model downstream to *Ngn*3, whereas we manipulated ROS signals upstream of *Ngn*3.

Contrary to the IUGR animals that display toxic oxidative stress in *β*-cells, ROS were found to increase insulin secretion in other models.^13,33^ For example, JunD−/− mice, which have a shortened lifespan, displayed hyperinsulinemia associated with increased pancreatic islet vascularization and chronic oxidative stress. Interestingly, long-term treatment of these mice with NAC from embryogenesis to adulthood restored blood glucose and plasma insulin, suggesting that antioxidant prevent insulin secretion.^33^ However, the treatment of wild-type (WT) mice with NAC did not alter glycemia or insulinemia.^33^ In this study of Laurent *et al.*,^33^ it would have been interesting to determine whether NGN3 expression varied in correlation with NAC supply. Moreover, the quantification of the *β*-cell mass at adulthood would allow to establish a link between ROS levels and the quantity of *β*-cells. Finally, *in vitro* experiments showed that incubation of rat islets with another antioxidant, trolox, blunted glucose-stimulated insulin secretion.^13^ Together, these data indicate that ROS, in addition to their role on *β*-cell development, have a crucial role on insulin secretion.

### ROS and MAPK signaling

In this study, H_2_O_2_ increased *β*-cell differentiation. As the half-life of H_2_O_2_ was only hours in the culture medium (Supplementary Figure S3), we expected to find a short-term activation of the signalization responsible for the upregulation of *Ngn*3 expression and observed that ERK1/2 activation occurred only 5 min after the addition of H_2_O_2_. Moreover, using an ERK1/2 inhibitor, we showed that an intact ERK1/2 pathway is necessary for the effects of H_2_O_2_ on endocrine differentiation. Interestingly, a link between ERK1/2 and *β*-cell development has been suggested previously. For example, an activation of the MAPK/ERK1/2 pathway by fibroblast growth factor-10 controls *β*-cell development.^19,34^ Moreover, in humans, *β*1-integrin/focal adhesion kinase activation of ERK1/2 is essential for human fetal islet maturation and survival.^35^ Such data reinforce our conclusion on the role of ERK1/2 in *β*-cell development, and indicate that the ERK1/2 pathway is controlled by different signals in the embryonic pancreas. Several studies have also shed light on the implication of ERK1/2 in the biology of differentiated *β*-cells. In the MIN6 *β*-cell line, ERK1/2 controls phosphorylation and protein level of cAMP-responsive element-binding protein (CREB), playing a key role in *β*-cell survival.^36^ Moreover, ROS are also required for insulin secretion in *β*-cells.^13^ Further studies will allow to establish the exact link between ROS and ERK1/2 in *β*-cell development and function.

### The effects of ROS on stem cell or progenitor cell differentiation are context dependent

Recent data highlighted the role of ROS in the differentiation of embryonic stem cells, induced pluripotent stem cells (iPS), and some adult stem cells, including hematopoietic stem cells or neural stem cells.^22, 37^ In these processes, both pluripotent and multipotent stem cells use enzymatic and nonenzymatic mechanisms for detoxification of ROS and for correction of oxidative damage to the genome or the proteome. Interestingly, a correlation exists between the antioxidant defense level and stem cell proliferation, differentiation, and death.^14^ Despite the broad influence of ROS-mediated signaling on cell differentiation, in many cases, cellular response to ROS depends on different cellular parameters, such as cell-type phenotype, cell differentiation state, and the presence of cell-specific transcription factors.^22^ Indeed, the variety of signals induced by H_2_O_2_ is wide. They include SIRT1 deacetylase, calcium influx, Nrf2, and PI3Kinase/Akt.^38, 39, 40^ Genotoxic stress caused by ROS may also induce the differentiation of cell types such as melanocytes.^41^ Thus, we cannot formally exclude that one of these processes is implicated in *β*-cell differentiation, in complement to ERK1/2 pathway. Further investigation will be performed to elucidate the role of other candidate pathways that may also exert an effect on *β*-cell differentiation in combination with ERK1/2.

In conclusion, we have identified a redox-mediated regulatory mechanism of differentiation that is required for *β*-cell development. This concept is counterintuitive. However, the possibility of a physiological role of ROS is in accordance with the findings showing that ROS are necessary for the function of *β*-cells.^13^ Moreover, it was recently postulated that type II diabetes is caused rather by a lack of biological oxidants than an excess of ROS.^42^ In this hypothesis, a failure of ROS could have wide effects, and be a cause of diabetes, cardiovascular disease, and some cancers. Our new data on the role of ROS during embryogenesis converge with this theory. In addition, our new findings also open perspectives for the generation of pancreatic *β*-cells from embryonic stem cells in order to develop a cell-based therapy of diabetes. Dissecting the defined roles of ROS in pancreatic progenitors will also greatly enhance their basic and translational applications.

## Materials and Methods

### Animals

Pregnant Wistar rats were purchased from the Janvier Breeding center (CERJ Janvier, Le Genest-Saint-Isle, France). The animals had free access to food pellets and water. Embryonic day 0.5 (E0.5) was considered the first day post coitum. Pregnant rats were killed by CO_2_ asphyxiation according to the guidelines of the French Animal Care Committee.

NAC (Sigma, Saint-Quentin Fallavier, France) treatment was initiated during embryonic life at E13.5 and given into drinking water to pregnant rats.

### Pancreatic dissection and culture

At E13.5, the dorsal pancreatic bud was dissected, as described previously.^34^ For the depletion of the mesenchyme, the digestive tract was incubated with 0.5 mg/ml collagenase A (Roche, Meylan, France) at 37°C for 30 min, then washed several times with Hank's balanced solution (Invitrogen, Cergy-Pontoise, France) at 4°C, and the epithelium was mechanically separated from the mesenchyme using needles on 0.25% agar, 25% Hank's balanced solution, and 75% RPMI gel (Life Technologies, Saint-Aubin, France) in a Petri dish. Pancreatic rudiments (epithelium with mesenchyme) or epithelia were then cultured at the air–medium interface on Millicell culture plate inserts (Millipore, Molsheim, France) in Petri dishes containing RPMI-1640 (Invitrogen) supplemented with penicillin (100 U/ml), streptomycin (100 g/ml), HEPES (10 mM), L-glutamine (2 mM), non-essential amino acids (1 × ; Invitrogen), and 10% heat-inactivated calf serum (Hyclone, South Logan, UT, USA) for 1 to 7 days. H_2_O_2_ (Sigma) was used at 25, 50, 75, and 100 *μ*M. Glucose (Sigma) was used at 20 mM, glucose oxidase (Sigma) at 0.3 *μ*/ml, and the inhibitor of the ERK pathway (U0126, Cell Signaling Technologies Ozyme, Saint-Quentin, France) at 10 mM. Cultures were maintained at 37°C in humidified 95% air and 5% CO_2_.

For proliferation measurement, BrdU (Sigma) was added during the last hour of culture (10 *μ*M).

For *β*-cell mass, pancreases were weighted. This weight was then multiplied by the corresponding *β*-cell fraction for each pancreas.

### Infection

Ad-CMV-GFP (ref 1060, Vector Biolabs, Philadelphia, PA, USA) and Ad-Catalase (ref 1475, Vector Biolabs) adenoviruses were used to infect the embryonic Wistar rat epithelia. Tissues were incubated with viral particles at the multiplicity of infection of 1000 for 4 h at 37°C in RPMI-1640 as described in Heinis *et al.*^43^ After infection, tissues were washed twice in HBSS and cultured at the air–medium interface.

### Immunohistochemistry

Tissues were fixed in 10% formalin and processed for immunohistochemistry, as described previously.^34^ The following antibodies were used: mouse anti-insulin (1 : 2000; Sigma-Aldrich, Saint-Quentin Fallavier, France), rabbit anti-glucagon (1 : 1000; Euromedex, Souffelweyerrsheim, France), rabbit anti-somatostatin (1 : 500; Dako, Glostrup, Denmark), rabbit anti-PDX1 (1 : 1000), rabbit anti-amylase (Sigma-Aldrich, 1 : 300), mouse anti-E-cadherin (1 : 100, BD Biosciences, Le Pont de Claix, France), mouse anti-BrdU (1 : 2, Amersham, Velizy-Villacoublay, France), rabbit anti-NGN3 (1 : 1000),^44^ rabbit anti-catalase (1 : 1000; Rockland Immunochemicals, Gilbertsville, PA, USA), rabbit anti-Phospho-p44/42 MAPK (Erk1/2) (Thr202/Tyr204) (1 : 200; Cell Signaling, Leiden, Netherlands), and rabbit anti-p44/42 MAPK (Erk1/2) (1 : 200; Cell Signaling). The fluorescent secondary antibodies were fluorescein isothiocyanate anti-rabbit and Texas Red anti-mouse antibodies (1 : 200, Jackson Immunoresearch, Suffolk, UK), and Alexa-fluor anti-rabbit antibody (1 : 400, Biogenex, Fremont, CA, USA). For NGN3, revelation was performed using the vectastain ABC kit (Vector LAB, Peterborough, UK). Fluorescent image acquisition was performed using an inverted fluorescence microscope Zeiss AxioObserver Z1 coupled with MRm Axiocam Zeiss (Zeiss, Marly le Roi, France).

### Insulin content

Pancreatic explants were cultured for 1 week. On day 7, pancreases were washed twice in Glucose-free HEPES-buffered Krebs-Ringer Buffer (116 mM NaCl, 5.06 mM KCl, 1.007 mM CaCl_2_, 1.01 mM MgCl_2_, 1.19 mM KH_2_PO_4_, 23.96 mM NaHCO_3_, 10 mM HEPES, pH 7.4, and 0.2% BSA). Pancreases were then collected in a tube and TetG was added. Tissues were then sonicated, centrifuged, and the supernatants were collected. Supernatants containing insulin were then tested using the Ultrasensitive Rat Insulin ELISA kit (Mercodia, Uppsala, Sweden) according to the manufacturer's instructions.

### Transferase-mediated dUTP nick-end labeling experiments

The TUNEL procedure was performed using an *in situ* cell death detection kit (Roche) according to the manufacturer's instructions. Subsequently, E-cadherin (1 : 100, BD Biosciences) immunostaining was used to visualize the epithelium.

### Real-time PCR

Total RNA was purified using the Rneasy microkit (Qiagen, Courtaboeuf, France). The cDNA was generated using Superscript reagents (Invitrogen), and the real-time PCR was performed on a 7300 real-time PCR system (Applied Biosystem, Life Technologies) with a SYBR Green PCR master mix. Cyclophilin A was used as housekeeping gene. The oligonucleotide sequences for RT-PCR are available on request.

### H_2_O_2_ stability measurement

Residual H_2_O_2_ was quantified by polarographic determination of the oxygen released upon addition of 10 *μ*g of purified beef liver catalase resuspended in 50 mM phosphate buffer pH 7.0 (Sigma) to 250 *μ*l culture medium samples placed in an oxygen polarograph (Hansatech, Norfolk, UK).

### Western blot analysis

For western blotting analysis, cells were lysed in Laemmli buffer. Proteins (20 *μ*g) were resolved by SDS-PAGE and electrophoretically transferred onto PVDF membrane (Bio-Rad, Hercules, CA, USA). After blocking with milk, membranes were probed with rat anti-p44/42 MAPK (Erk1/2) (Cell Signaling) and mouse anti-Phospho-p44/42 MAPK (Erk1/2) (Thr202/Tyr204, Cell Signaling). Immunoreactive bands were visualized with the SuperSignal System (Pierce, Fisher Scientific, Illkirch, France).

### Quantification

To quantify the absolute number of insulin-expressing cells, all sections of each pancreatic rudiment were digitized. On every image, the surface area of insulin staining was quantified using ImageJ (NIH, Washington, WA, USA) as described previously.^43^

To quantify the number of NGN3-positive cells, cells immunopositive for NGN3 were counted on all sections. Statistical significance was determined using Student's *t-*test.

To measure the proliferation of early progenitors expressing E-cadherin, we counted the frequency of BrdU^+^ nuclei among 1000 PDX1^+^ cells. Three rudiments per condition were analyzed. Statistical significance was determined using Student's *t-*test.

To measure cell survival, we counted the frequency of TUNEL^+^ cells among E-cadherin^+^ cells. Statistical significance was determined using Student's *t-*test.

## Abbreviations

Ad-Cat: adenovirus coding for catalase
Ad-GFP: adenovirus coding for GFP
CREB: cAMP-responsive element-binding protein
CCCP: carbonyl cyanide *m*-chlorophenyl hydrazone
GOx: glucose oxidase
GPx: glutathione peroxidase
HO-1: heme oxygenase
H_2_O_2_: hydrogen peroxide
iPS: induced pluripotent stem cells
IUGR: intrauterine growth retardation
MAPK: mitogen-activated protein kinase
NAC: *N*-acetyl-cysteine
NGN3: neurogenin 3
Nrf2: nuclear-factor E2 related factor 2
PDX1: pancreatic and duodenal homeobox 1
PKC: protein kinase C
ROS: reactive oxygen species
WT: wild type
