# Supplementary material for: Pro-oxidant/antioxidant balance controls pancreatic β-cell differentiation through the ERK1/2 pathway
Source: Cell Death Dis. 2014 Oct 23;5(10):e1487–. doi: 10.1038/cddis.2014.441 (PMC4237262; doi:10.1038/cddis.2014.441)
Supplement: Supplementary Figure S1 [file cddis2014441x1.pdf]

	Adult Liver	Adult Pancreas		E13.5 Embryonic Pancreas	
		Percentage	*p value*	*Percentage*	*p value*
Catalase	100	**2.45**	$1.4 .10^{-4}$	**0.81**	$1.3 .10^{-4}$
Gluthathion Peroxidase	100	**12.88**	$4.2 .10^{-3}$	**2.82**	$2.7 .10^{-3}$

Expression of genes encoding for ROS-scavenging enzymes in adult and embryonic tissues
