# Supplementary figures and images for: Pro-oxidant/antioxidant balance controls pancreatic β-cell differentiation through the ERK1/2 pathway
Source: Cell Death Dis. 2014 Oct 23;5(10):e1487–. doi: 10.1038/cddis.2014.441 (PMC4237262; doi:10.1038/cddis.2014.441)

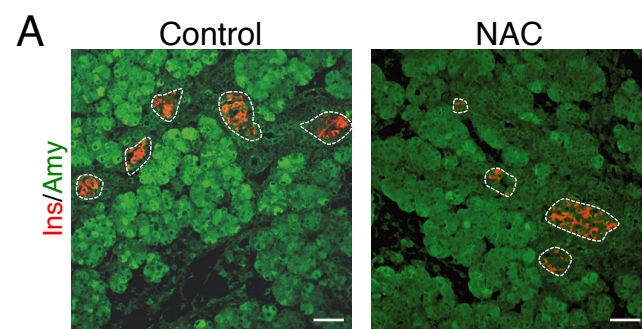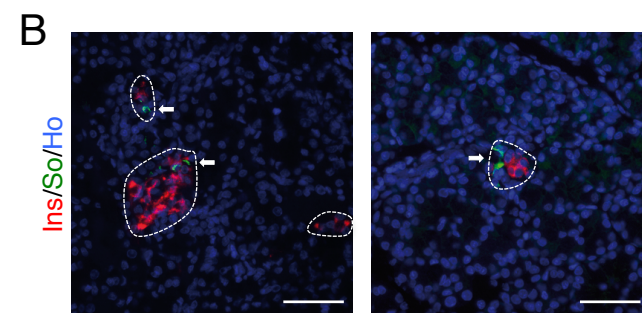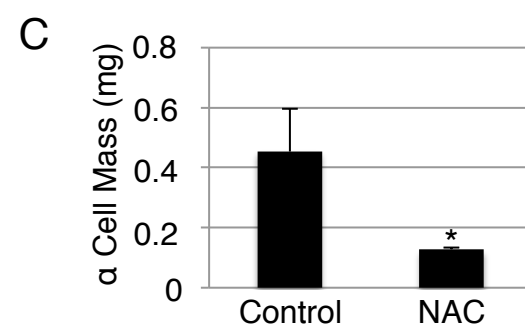

Supplement: Supplementary Figure S2 [file cddis2014441x2.pdf]

A

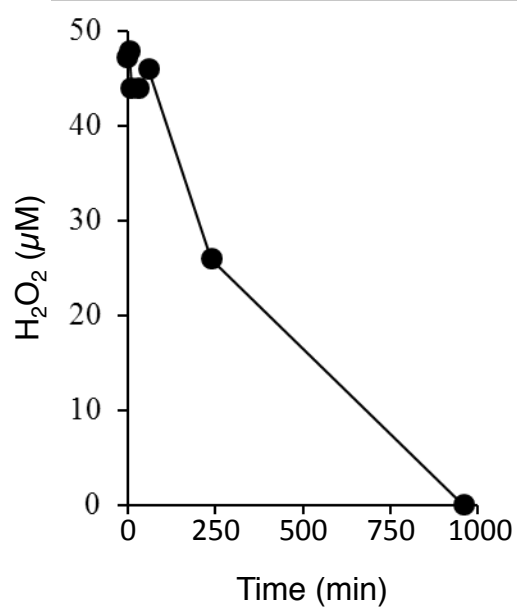

B

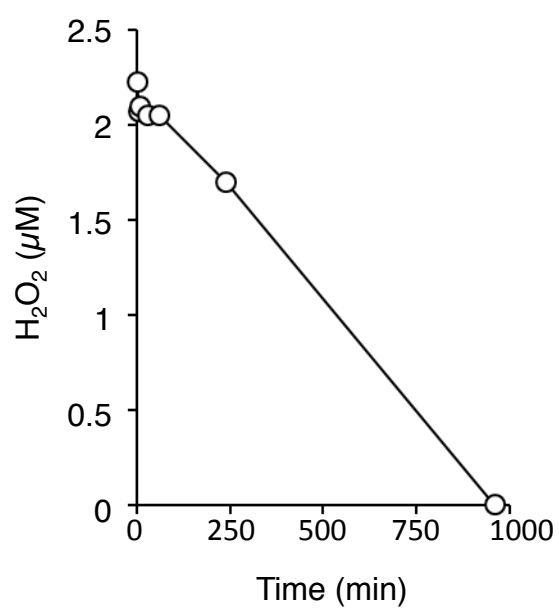

Supplement: Supplementary Figure S3 [file cddis2014441x3.pdf]

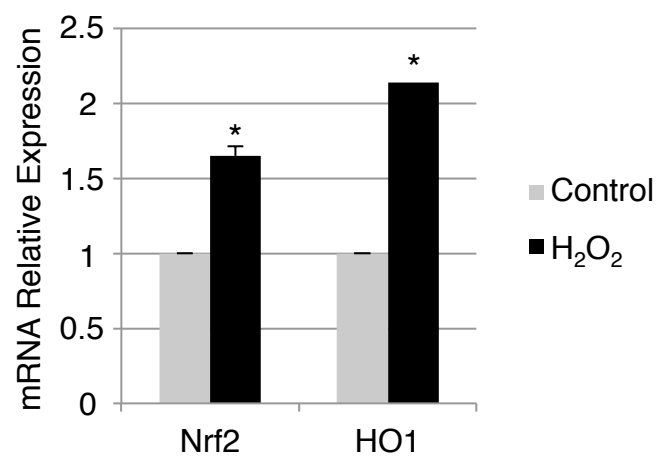

Supplement: Supplementary Figure S4 [file cddis2014441x4.pdf]

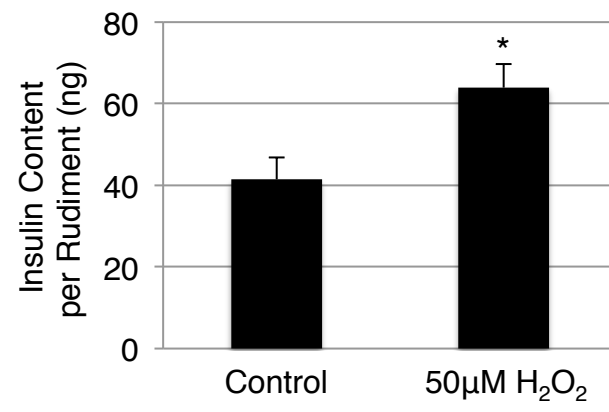

Supplement: Supplementary Figure S5 [file cddis2014441x5.pdf]

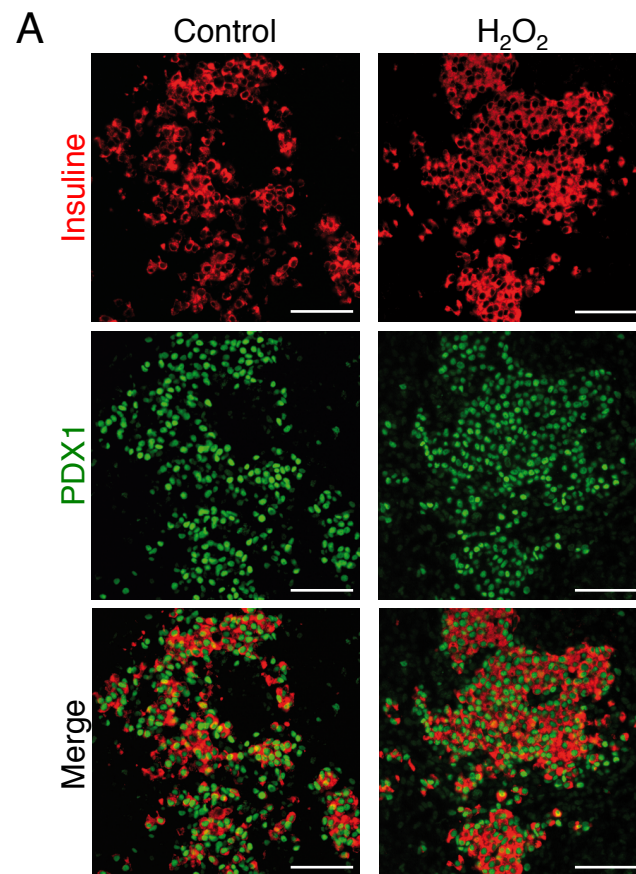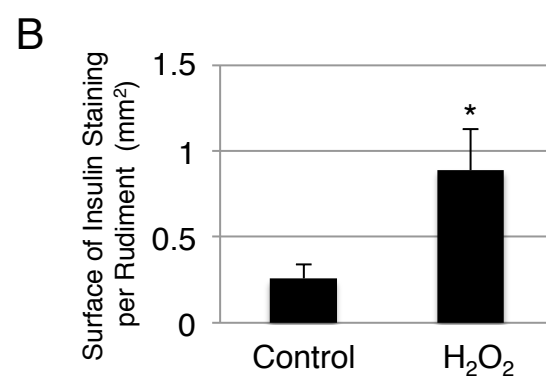

Supplement: Supplementary Figure S6 [file cddis2014441x6.pdf]

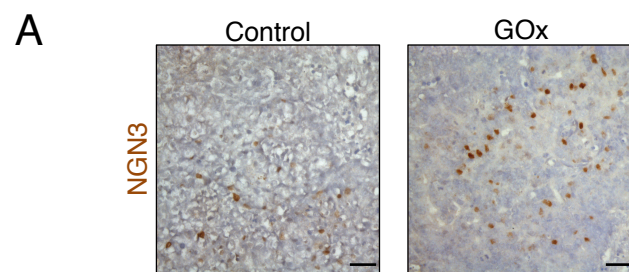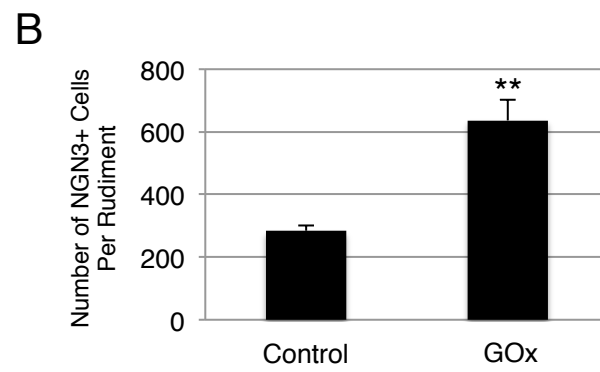

Supplement: Supplementary Figure S7 [file cddis2014441x7.pdf]

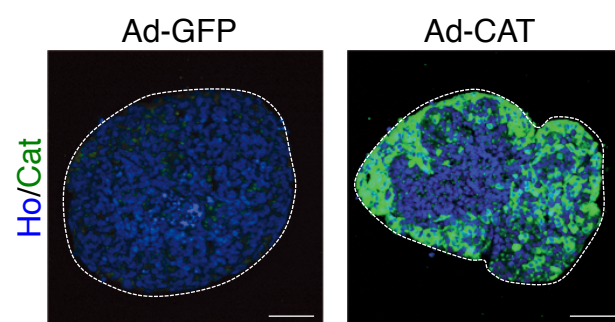

Supplement: Supplementary Figure S8 [file cddis2014441x8.pdf]

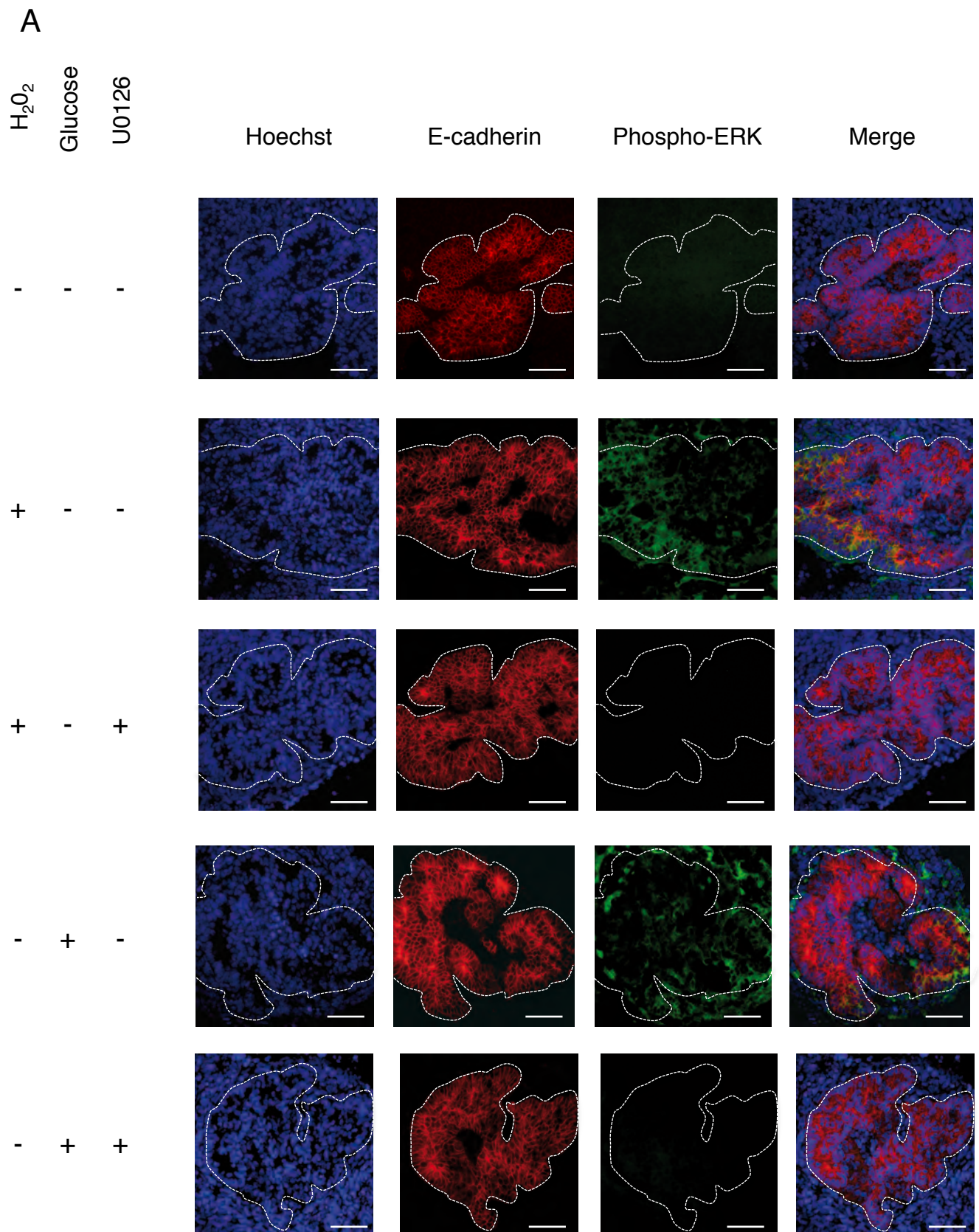

B

H<sub>2</sub>O<sub>2</sub>  
Glucose  
U0126

Hoechst

E-cadherin

ERK

Merge

- - -

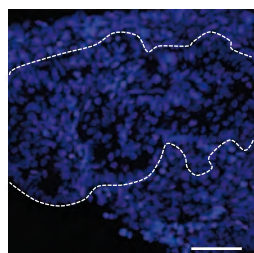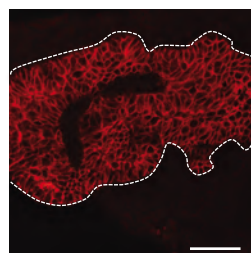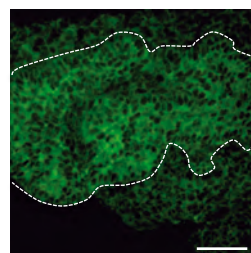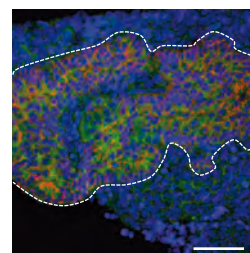

+ - -

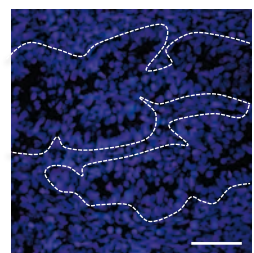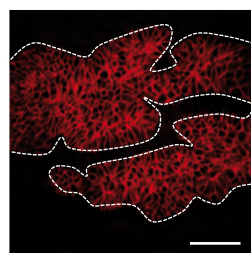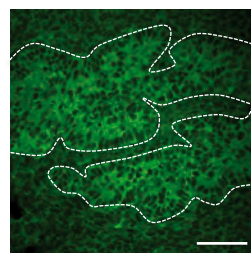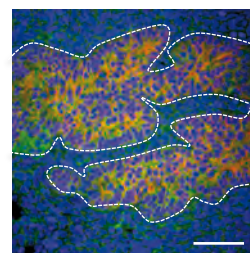

+ - +

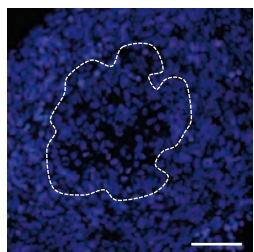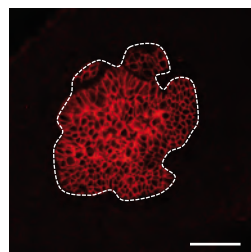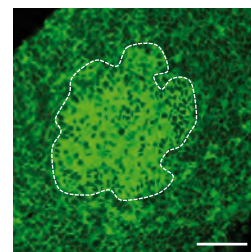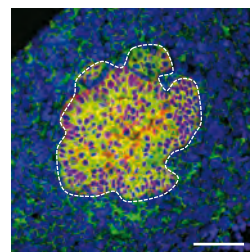

- + -

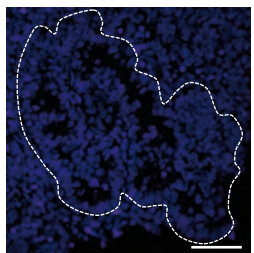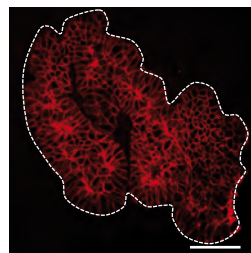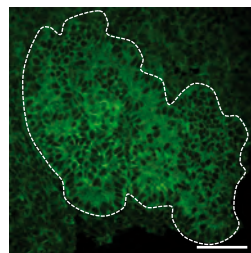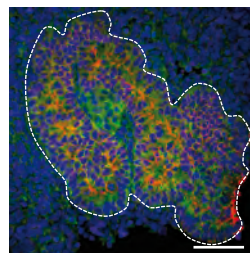

- + +

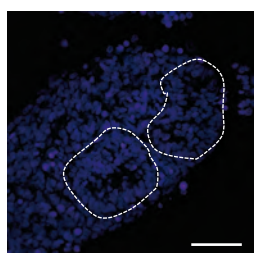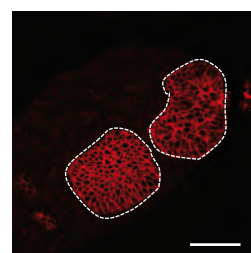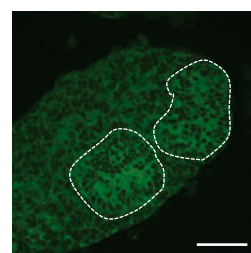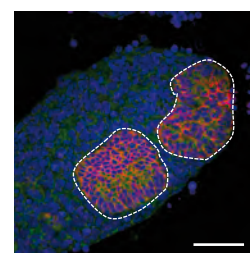

Supplement: Supplementary Figure S9 [file cddis2014441x9.pdf]
