# Supplementary material for: Pro-oxidant/antioxidant balance controls pancreatic β-cell differentiation through the ERK1/2 pathway
Source: Cell Death Dis. 2014 Oct 23;5(10):e1487–. doi: 10.1038/cddis.2014.441 (PMC4237262; doi:10.1038/cddis.2014.441)
Supplement: Supplementary Figure Legends [file cddis2014441x10.doc]

**Supplemental Data.**

SUPPLEMENTARY 1. *Antioxidant enzyme expression in adult and embryonic pancreas and liver.*

E.13.5 embryonic and adult pancreases and livers were dissected and total RNA were extracted and purified. A real-time PCR was then runned to detect the expression of the antioxidant enzyme Catalase and Gluthation Peroxidase. Values are expressed at the percentage of the adult liver gene expression. Each value represents the mean ± SEM of three individual data pools.

SUPPLEMENTARY 2. *NAC treatment does not affect pancreas morphology but decreases α-cell mass.*

Pregnant rats were treated with 10 mM NAC from 13.5 days post-coïtum. Embryonic pancreases were analyzed at E20.5. A : β-cells and acinar tissue were dectected using anti-insulin (red) and anti-amylase (green) respectively. Nuclei were stained with Hoechst 33342 (blue). B : β-cells (red) and δ-cells (green) were detected using anti-insulin and anti-somatostatin antibodies respectively. Nuclei were stained with Hoechst 33342 (blue). C : α-cells were detected using anti-glucagon antibodies and glucagon staining was quantified. α-cell mass was then calculated. Each point represents the mean ± SEM of three individual data pools. *P <0.05. Scale bar : 50 μm.

SUPPLEMENTARY 3. *Kinetics of hydrogen peroxide decay at 37°C in culture medium.*

Residual hydrogen peroxide (initial concentration: A : 50 μM, B : or 2.5 μM) was quantified in the culture medium by a polarograph.

SUPPLEMENTARY 4. *Expression of Nrf-2 and HO-1 are increased by ROS stimulation.*

E13.5 pancreases were cultured for 24 hours with or without H_2_O_2_ and Nrf-2 and HO-1 mRNA levels were quantified by qPCR. *P <0.05.

SUPPLEMENTARY 5. *Insulin content after ROS treatment.*

Insulin content from pancreatic explants cultured with or without H_2_O_2_ in static cultures for one week. Each point represents the mean ± SEM of three individual data pools. ***P < 0.001.

SUPPLEMENTARY 6. *H_2_O_2_ increases the number of mature β-cells.*

A : E13.5 rat pancreases were cultured for 7 days with or without H_2_O_2_ (50 μM). At day 7, antibodies directed against insulin (red) and PDX1 (green) were used to detect differentiated β cells. B : The absolute surface area occupied by insulin-positive cells was quantified. All cells marked for insulin were also positive for PDX1. Each point represents the mean ± SEM of three individual data pools. *P <0.05; Scale bar : 50 μm.

SUPPLEMENTARY 7. *Glucose oxidase mimics the effect of ROS on* ß-*cell development.*

Pancreases were cultured one day with GOx at 0,3U/mL. A : NGN3 expression (in brown) was detected by immunohistochemistry. B : The number of NGN3-positive cells was then quantified for each rudiment. Each point represents the mean ± SEM of three individual data pools. **P < 0.01. Scale bar : 25 μm.

SUPPLEMENTARY 8. *Infection of pancreatic epithelia with Ad-Cat increases catalase expression.*

Pancreatic epithelia were infected with adenoviruses coding either for GFP or for catalase. After one day, catalase (in green) was detected by immunohistochemistry. Nuclei were stained with Hoechst 33342 (blue). Scale Bar: 50 μm.

SUPPLEMENTARY 9. *Phosphorylation of ERK1/2 in pancreases cultured with or without H_2_O_2_ or Glucose.*

Pancreases were cultured 5 min with or without 50 μM H_2_O_2_ or 20mM Glucose. A : ERK1/2 Phosphorylation and epithelium were then detected by immunohistochemistry using anti-Phospho-ERK1/2 and anti-E-cadherin antibodies. B : In the same, culture conditions, total ERK1/2 was also detected. Scale bar : 50 μm.
